# Pro-Inflammatory S100A8 and S100A9 Proteins: Self-Assembly into Multifunctional Native and Amyloid Complexes

**DOI:** 10.3390/ijms13032893

**Published:** 2012-03-05

**Authors:** Thomas Vogl, Anna L. Gharibyan, Ludmilla A. Morozova-Roche

**Affiliations:** 1 Institute of Immunology, University of Muenster, Röntgenstr. 21, 48149 Muenster, Germany; E-Mail: vogl@uni-muenster.de; 2 Department of Medical Biochemistry and Biophysics, Umea University, SE-901 87 Umea, Sweden; E-Mail: anna.gharibyan@medchem.umu.se

**Keywords:** S100A8, S100A9, S100 proteins, amyloid, inflammation, cancer, self-assembly, calcium-binding, calprotectin

## Abstract

S100A8 and S100A9 are EF-hand Ca^2+^ binding proteins belonging to the S100 family. They are abundant in cytosol of phagocytes and play critical roles in numerous cellular processes such as motility and danger signaling by interacting and modulating the activity of target proteins. S100A8 and S100A9 expression levels increased in many types of cancer, neurodegenerative disorders, inflammatory and autoimmune diseases and they are implicated in the numerous disease pathologies. The Ca^2+^ and Zn^2+^-binding properties of S100A8/A9 have a pivotal influence on their conformation and oligomerization state, including self-assembly into homo- and heterodimers, tetramers and larger oligomers. Here we review how the unique chemical and conformational properties of individual proteins and their structural plasticity at the quaternary level account for S100A8/A9 functional diversity. Additional functional diversification occurs via non-covalent assembly into oligomeric and fibrillar amyloid complexes discovered in the aging prostate and reproduced *in vitro*. This process is also regulated by Ca^2+^and Zn^2+^-binding and effectively competes with the formation of the native complexes. High intrinsic amyloid-forming capacity of S100A8/A9 proteins may lead to their amyloid depositions in numerous ailments characterized by their elevated expression patterns and have additional pathological significance requiring further thorough investigation.

## 1. Introduction

S100A8 and S100A9 proteins became the focus of intensive current research due to their association with numerous human disorders, including acute and chronic inflammatory conditions, autoimmune diseases, cancer, atherosclerosis, cardiomyopathies and neurodegenerative diseases [1–5], as well as due to their crucial roles in normal physiological processes within cells. Apparently these proteins are able to perform a wide plethora of intra- and extracellular functions, including cytokine-like and chemokine-like activities via activation of the receptor for advanced glycation end products (RAGE) and Toll-like receptor 4 (TLR4) dependent signaling cascades and potentially other signaling pathways, promotion of calcification in the blood vessels and prostate, regulation of cytoskeleton via tubulin polymerization and others [6]. In the course of on-going research we may expect that new functions will be discovered. Such remarkable multiple functionality gives rise to the question—which structural properties can underlie this phenomenon? The structural and functional diversity of the whole S100 family including its *ca.* 22 members was discussed in recent review [7]. Here we shed light on how the structural plasticity of S100A8 and S100A9 and their ability to self-assemble into various native and amyloid complexes can lead to their highly diversified activities.

S100A8 and S100A9 belong to the family of low molecular weight S100 proteins (10–13 kDa) comprising 22 members to date and representing the largest subfamily of the EF-hand Ca^2+^-binding proteins [8]. All S100 proteins share conserved structural motifs of two EF-hand Ca^2+^-binding domains connected by a variable hinge region that often confer biological activity [9]. The tendency to form homodimers is common to all S100 proteins, including S100A8 and S100A9, with one exception—calbindin D9k is monomeric [10]. Some members of the S100 protein family are also able to form heterodimers as observed f.e. for S100A8 and S100A9 or S100A1 and S100P, which suggests different functions for homo- and heterodimers. The conformation and stability of S100A8 and S100A9 are drastically modulated by metal ion binding. The binding of Ca^2+^ to EF-hand type domains triggers conformational movements allowing interactions with other proteins. The binding of Zn^2+^ additionally modulates structural properties of these proteins [11,12]. As a result, S100A8 and S100A9 heterodimers assemble in a Ca^2+^ and Zn^2+^-dependent manner into heterotetrameric and larger complexes [13,14]. Once formed, the heterotetrameric S100A8/A9 complexes are highly stabilized compared to their hetero- and/or homodimers and can fulfill other functional properties absent in the monomeric and dimeric precursors. Recently a new property of S100A8 and S100A9 was discovered—their ability to self-assemble into highly heterogeneous amyloid complexes, encompassing both oligomeric species and highly stable fibrils able to grow and accumulate in the ageing prostate [15]. This fact adds up to the structural and functional diversity of S100A8/A9 self-assembled complexes and their multiple roles in health and disease discussed below.

## 2. Nomenclature

S100A8 and S100A9 as well as other S100 proteins are found only in vertebrates. The S100 proteins were first named by Moore according to their solubility in 100% saturated ammonium sulfate [16]. However, the nomenclature of S100A8 and S100A9 includes a few additional names. As their expression was discovered in cells of myeloid origin, namely macrophages infiltrating inflamed tissues, they were initially coined as migration inhibitory factor-related proteins but later renamed as myeloid-related proteins, *i.e.*, MRP-8 and MRP-14, respectively [17–19]. In addition S100A8 is denoted also as calgranulin A and S100A9 as calgranulin B [20], reflecting their calcium-binding properties and high expression in granulocytes. The heterotetrameric S100A8/A9 complex is known as calprotectin [21]. S100A8 was the first S100 family member described as having potent chemotactic activity for murine neutrophils and monocytes and was initially named CP10—chemotactic protein 10 kDa, to reflect its function and molecular mass [22]. Here we will use the S100A8 and S100A9 nomenclature as the most established to date and updated for the whole S100 family in the review [23].

## 3. Primary Structure and Ca^2+^-Binding Sites

S100A8 and S100A9 are proteins with molecular masses of 10.8 and 13.2 kDa and compositions of 93 and 114 amino acids, respectively. All S100 proteins possess highly conserved overall structural architectures, although their sequences are homologous not more than by 25–65% [24]. Similar to other S100 proteins S100A8 and S100A9 contain two Ca^2+^-binding sites of the “EF-hand type”, denoting a helix-loop-helix motif involved in Ca^2+^ coordination [25]. The far UV CD spectrum of S100A8 and S100A9 indicates a high degree of α-helical content, which is characteristic for the EF-hand protein family [26,27].

**Figure 1:**
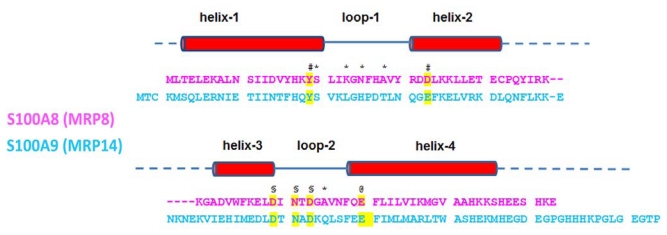
Aligned amino-acid sequences of S100A8 and S100A9 with schematically highlighted α-helices and Ca^2+^-binding loops (Adapted from Ishikawa *et al*. [28]). The conserved residues that side chains bind Ca^2+^ are shown in yellow. Groups coordinating Ca^2+^ are indicated as follow: * backbone carbonyl groups; ^#^ water-mediated; ^§^ monodentate side chain of Asp or Asn; ^@^ bidentate side chain of Glu.

The *C*-terminal canonical EF-hand (site II) has a typical 12 amino acid residue Ca^2+^-binding loop positioned between two supporting α-helices (Figure 1) [28,29]. In the case of S100A8 the Ca^2+^-binding site includes residues Asp58–Glu70 and in S100A9—residues Asp67–Glu78. Seven oxygen ligands interact with Ca^2+^, forming a pentagonal bipyramidal coordination: in the case of S100A8 these are the monodentate Asp59, Asn61 and Asp63, the bidentate Glu70, the main-chain carbonyl group of Ala65 and a water molecule [28]; in the case of S100A9—the side-chain O atoms of Asp67, Asn69, Asp71 and Glu78, the main-chain carbonyl O atom and a water molecule [29] (as indicated in Figure 1). However, the *N*-terminal pseudo EF-hand (site I) possesses an extended EF-hand loop with 14 amino acid residues, which is a distinctive feature of the S100 family and characterized by the lower affinity towards Ca^2+^. In the case of S100A8 these are residues Ser20–Asp33 and in the case of S100A9—residues Ser23–Glu37. The alignment of the Ca^2+^-binding motifs of S100A8 and S100A9 with other members of the S100 family shows that in both sites S100A9 contains the conserved sequence determinants necessary for Ca^2+^-binding. Specifically in the site I of S100A9 seven O atoms from the main-chain carbonyl groups of Ser23, Leu26, His28 and Thr31, the carboxyl group of Glu36 and a water molecule bind Ca^2+^ [29] as indicted in Figure 1. By contrast, in S100A8 four main-chain carbonyl groups of Ser20, Lys23, Asn25 and Ala28 make four ligands to the central Ca^2+^ [28]. In the conserved EF-hand the fifth ligand is a side-chain O atom of glutamic acid (Figure 1), while in S100A8 the corresponding residue was replaced by Asp33, which has a shorter side chain. Because of this change, the O atom of Asp33 is not directly ligated to Ca^2+^, but a water molecule can bridge the O atom of Asp33 and the Ca^2+^ [28]. There is no sixth ligand in this site, which is usually occupied by a water molecule. Glutamic acid is highly conserved in this position in S100 proteins and in the EF-hand Ca^2+^-binding protein family as a whole [30]. This residue is known to play an important role in Ca^2+^-binding by providing a bi-dentate ligand to the Ca^2+^ ion and forming an integral part of a network of hydrogen bonds that stabilize the binding loop. For example, the substitution of bidentate Ca^2+^-coordinating Glu14 drastically reduces Ca^2+^-affinity and alters the large Ca^2+^-induced conformational changes occurring in the Ca^2+^-sensoring proteins such as calmodulin and troponin C [31,32], which implies that the Glu33Asp substitution in site I of S100A8 may be critical for its Ca^2+^-affinity and functionality. It has been shown that when S100A8 and S100A9 self-assemble into the complexes, their dimer binds 4 Ca^2+^ and tetramer—8 Ca^2+^ ions, respectively [14].

Both *N* and *C*-terminal sequences of these proteins are relatively hydrophobic and the *C*-terminus of S100A9 is the longest within the family (Figure 1). It is interesting to note, that two main isoforms are known for S100A9 purified from granulocytes—the full length and truncated proteins, S100A9 (13.2 kDa) and S100A9* (12.7 kDa), respectively. The latter is shorter by the first four *N*-terminal amino acids due to alternative translation [14]. As a result, upon different combinations of these isoforms, two types of heterodimeric and three types of heterotetrameric complexes of S100A8/A9 can be isolated from granulocytes [14].

S100A8 and S100A9 each contains single cysteine and tryptophan residues. S100A9 is phosphorylated in Ca^2+^-dependent manner at the *C*-terminal site of Thr113 [33], which increases reciprocally its Ca^2+^-affinity and favors its translocation from cytosol to the cytoskeleton and membrane [34,35]. We have identified that p38 kinase is responsible for S100A9 phosphorylation [6,36]. The differential post-translational modifications of S100A9 suggest a possible distinct function for each of their isoforms, which can also add up to their inherent multiple functionalities.

## 4. Zn^2+^-Binding

Besides Ca^2+^, S100A8 and S100A9 also bind Zn^2+^ ions. Both proteins contain HEXXH motives in their sequences that are putative Zn^2+^-binding sites [37]. It has been shown that extracellular S100A8/A9 exhibits antimicrobial activity just by chelation of Zn^2+^ ions [37], which is necessary for bacterial growth. At the same time neither S100A8 nor S100A9 tested alone showed antimicrobial activity. Only when both proteins are used in combination, Zn^2+^-reversible activity takes place, suggesting that the heterodimer is necessary to form a Zn^2+^-binding site capable of inhibiting microbial growth. The Zn^2+^-induced S100A8/A9-tetramerization was also demonstrated by using biophysical methods such as MALDI-MS, ESI-MS and fluorescence spectroscopy [13,38]. In these experiments Zn^2+^ triggered the formation of S100A8/A9 heterotetramers by binding to both Ca^2+^-specific EF-hands and by Zn^2+^-specific binding sites and the Zn^2+^-induced tetramer was structurally very similar to the Ca^2+^-induced one. We observed 12 Zn^2+^ bound to the tetramer (8 Zn^2+^ were coordinated by Ca^2+^-specific EF-hands and 4 Zn^2+^—by the Zn^2+^-specific binding sites). Consequently, after addition of Ca^2+^ 8 out of the 12 Zn^2+^ were replaced by Ca^2+^ apparently via binding to the Ca^2+^-specific EF-hand binding sites [13,38].

The X-ray diffraction analysis of human S100A8/A9 heterotetramer—calprotectin revealed that two different putative Zn^2+^-binding sites occur at the S100A8/A9 subunit interface [39]. One of these corresponds to a high affinity arrangement of three His residues and one Asp side-chain, which is unique to the heterotetramer [37]. Specifically, in the heterotetramer His83 and His87 from the HXXXH motif of S100A8 are complemented by His20 from the opposite S100A9 molecule. Together with the side-chain of Asp30 in spatial neighborhood this would allow the formation of a His_3_Asp Zn^2+^ binding motif [39]. In addition, the homologous residues His91 and His95 from the HXXXH motif of S100A9 can be complemented by His17 and His27 from S100A8 rather than by Asp, to be involved in tetrahedral metal coordination by His_4_Zn^2+^ binding site [39]. Thus, Ca^2+^, Zn^2+^ and possibly other cations can also act as regulatory factors in S100A8/A9 assembly and functions.

Noticeably, Zn^2+^-binding induces different functional properties compared to Ca^2+^, for example, Zn^2+^ reverses the Ca^2+^-induced arachidonic acid binding by the S100A8/A9 complex [40]. The arachidonic acid binding was not induced by the coordination to the protein complex of Zn^2+^, Cu^2+^ and Mg^2+^. In contrast, the binding of arachidonic acid was prevented by the addition of either Zn^2+^ or Cu^2+^ in the presence of Ca^2+^, whereas Mg^2+^ failed to abrogate this property. The inhibitory effect was not due to the blocking of S100A8/A9 complex formation, but the fluorescence measurements provided evidence that both Zn^2+^ and Cu^2+^ induce different conformational changes affecting the Ca^2+^-induced formation of the arachidonic acid binding pocket within the S100A8/A9 complex. As Zn^2+^ produces the inhibitory effect at physiological serum concentrations, this suggests that released S100A8/A9 may carry arachidonic acid at inflammatory lesions, but not within the blood compartment. Moreover, acting as a host Zn^2+^-chelator, the S100A8/A9 heterocomplex—calprotectin is able to carry out the function of a neutrophil-associated antifungal agent when it is expressed within neutrophil extracellular traps (NETs) and reversibly prevents *Aspergillus nidulans* growth at low concentrations, while leads to irreversible fungal starvation at higher concentrations [41]. Calprotectin was suggested also to act as a chelator of manganese and zinc ions in the host defence against *S. aureus* infection [42].

It is interesting to note, that the biological activity of other S100 proteins is also regulated by Zn^2+^ as well as by Ca^2+^ [7]. The Zn^2+^-binding sites of S100B, S100A2, S100A7 and S100A12 were characterized in detail by using mutational, NMR and crystallographic analyses [43–46]. These S100 proteins bind two Zn^2+^-ions per homodimer and the Zn^2+^-binding sites are formed by residues from both subunits. Since the Zn^2+^-binding residues in S100A9 and S100A7 are fully conserved, it is likely that the S100A8/A9-dimer coordinates Zn^2+^ in a similar way as S100A7 [39,45,47]. Similarly, the Zn^2+^ binding regulates the interactions of other S100 proteins with their target molecules such as RAGE, tau and others, illustrating a cross-talk between Ca^2+^ and Zn^2+^ in modulating protein conformations for specific functional properties [7,46,48].

## 5. Dimerisation and Tetramerisation

**Figure 2:**
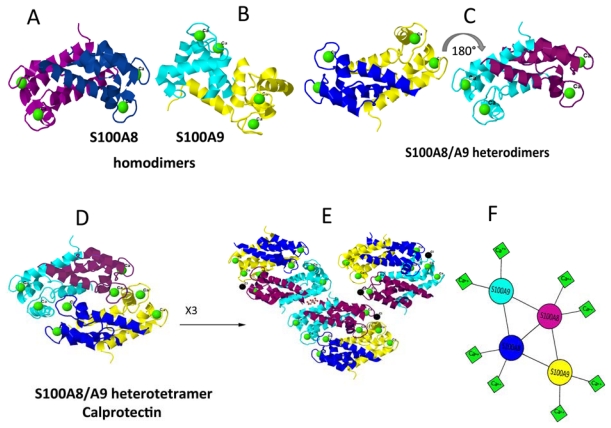
Tertiary and quaternary structures of S100A8 and S100A9 proteins presented by ribbon diagrams: Source of PDB files [56]: A [57], B [58], C–E [59]. (**A**) S100A8 homodimer; individual subunits are shown in purple and dark blue; (**B**) S100A9 homodimer; subunits are shown in sea-blue and yellow; (**C**) S100A8/A9 heterodimers shown in two projections rotated by 180°; (**D**) S100A8/A9 heterotetramer calprotectin and (**E**) S100A8/A9 dodecamer assembled from 3 calprotectins; (**F**) Schematic outline of the arrangements of S100SA8 and S100A9 in calprotectin. Subunits are presented in individual colors as in (**A**) and (**B**). Bound Ca^2+^ ions are shown by green spheres or squares, respectively. The images A–E are created with Jmol [60].

Both S100A8 and S100A9 form stable non-covalently associated homo- and heterodimers [28,29] (Figure 2A–C). The heterocomplex is usually purified from neutrophils. Circulating neutrophils and monocytes represent the first cells to invade inflammatory lesions. The heterodimeric complex is the predominantly occurring form and considered as physiologically relevant [6,14,49,50]. However, recent results also showed that homodimers of S100A8 and S100A9 exhibit strong pro-inflammatory and cytokine-inducing activities in various mouse models of disease [51–53]. It should be noted, that currently no method is able to determine the fractions of homo- and heterodimers and to discriminate between homo- and heterodimeric effects if all three dimeric species are present simultaneously. The dissociation constants of the homo- and heterodimers are not defined yet and although the heterodimers are certainly more stable than the homodimers, all three species can exist in parallel [54]. The microcalorimetric heat denaturation studies suggest a lower stability of S100A9 homodimer compared to that of S100A8 in the absence of calcium. Ca^2+^-binding dramatically increases the thermal stability of both homodimers as well as of the heterodimer [38,39].

The heterodimer is essential for S100A8 and S100A9 tetramerisation [38]. The association into S100A8/A9 heterotetramer occurs upon Ca^2+^-binding, that the final complex binds 8 Ca^2+^ (Figure 2D,F) [13,14,26,55]. This fact was demonstrated also by mutation analysis of S100A9 (Figure 2B) [27,38]. The mutations were introduced in the *C*-terminal EF-hand of S100A9, substituting ligands essential for Ca^2+^-coordination by alanine—Asn69Ala, Glu78Ala and double mutations Asn69Ala + Glu78Ala. The Ca^2+^-regulated heterotetramer formation was strictly dependent on the functional EF-hand II in S100A9 as revealed by density gradient centrifugation and mass spectrometry. As the Ca^2+^-binding capacity of S100A9 mutants was diminished, the functional properties of S100A8/A9 complexes were also altered as reflected in their effect on tubulin polymerization. Despite the diminished Ca^2+^-binding capacity of S100A9 mutants, the heterodimers containing the Asn69Ala and Glu78Ala mutants of S100A9 were still able to interact with tubulin filaments in a Ca^2+^-dependent manner, reflecting that this function was mediated via S100A8 [6,27]. However, the polymerization rate of tubulin in the presence of S100A9 mutants was significantly decreased, indicating the essential role of heterotetramer in this process.

The 1.8 Å crystal structure of S100A8/A9 also revealed that tetramerization is the product of dimerization of heterodimers S100A8/A9 [39]. Specific heteroassociation is energetically driven by an extensive burial of solvent accessible surface areas of both proteins and particularly S100A9 within the heterotetramer quaternary structure (Figure 2D). The canonical Ca^2+^-binding loops in the *C*-terminals (sites II) of both proteins are major contributors to heterotetramer association. The mode of heterodimerization more closely resembles the subunit association observed in the S100A8 homodimer and provides trans-stabilization for S100A9, which leads to a significantly elongated *C*-terminal α-helix in the latter.

In contrast to human S100A8 and S100A9 complexes, much less is known about murine S100A8 and S100A9 homo- and heterodimers. By using yeast two-hybrid studies it has been shown that the murine homo- and heterodimers exist in parallel [55]. Investigating the S100A8 and S100A9 oligomer formation by size exclusion chromatography, we observed that neither the homodimers of both human and murine proteins nor the murine heterodimers are able to form higher molecular oligomers [61]. These findings suggest that the oligomer formation is a unique feature for the human S100A8/A9 heterodimers.

## 6. Clinical Occurrence and Functional Diversity

To date there is growing evidence that S100A8 and S100A9 are involved in numerous biological processes both intra- and extracellular. This plethora of functions lies in their ability to interact with various proteinaceous targets and to modulate their structures and functions. At the same time S100A8 and S100A9 lack their own enzymatic activity. As discussed above, the conformation and dimerization/tetramerization of S100A8/A9 are responsive to Ca^2+^ as well as to Zn^2+^ and possibly other metal ions. Therefore, they can mediate Ca^2+^ signals by binding to other target proteins and modulating their conformation and activity in a Ca^2+^ or other metal ion dependent manner. The assembly into homo- and heterodimeric, tetrameric and even larger oligomeric complexes is also very versatile generic mechanism of protein structural and consequently functional diversification, which can also underlie the multiple roles of S100A8 and S100A9 in various tissues and organs presented in this chapter.

### 6.1. S100A8 and S100A9 in Myeloid Cells

S100A8 and S100A9 are mainly expressed in cells of myeloid origin, such as granulocytes, monocytes and early stages of macrophages, but not in resident tissue macrophages. They constitute up to 40% of neutrophil cytosolic protein (5% in monocytes), suggesting that they perform a key role in the activities of these cells [62]. S100A8 and S100A9 are also expressed in keratinocytes and epithelial cells but only under inflammatory conditions [63]. For a long time it has been thought, that S100A8/A9 act as calcium buffers, calcium sensors and/or intracellular differentiation factors for phagocytes. But none of these proposed functions could be verified. Indeed, only minor differences in intracellular calcium signaling of the phagocytes derived from S100A9 −/− mice were observed compared to wild-type cells [64]. Furthermore, the myelopoietic potential of the S100A9 −/− bone marrow was normal, suggesting that a deficiency in S100A9 was compatible with viable and mature phagocytes [65,66].

Among intracellular functions of S100A8 and S100A9, the calcium-dependent interactions of S100A8/A9 complex with cytoskeletal components are well established. Specific binding of S100A8/A9 to microtubules, vimentin, keratin and actin filaments were described [6,36,49,67,68]. However, functional correlations were found only for the interactions of hetero-S100A8/A9 complexes to microtubules in human monocytes [6] and to actin filaments in human neutrophils [36]. Lominadze *et al*. observed the association of S100A8/A9 complexes with actin filaments and confirmed that S100A9 phosphorylation by p38 mitogen-activated protein kinase (MAP kinase) takes place also in human neutrophils [36], however, the functional consequences of these findings remain unclear. S100A8/A9 promotes tubulin polymerization and bundling of microtubules in a strictly Ca^2+^-dependent manner [6] and the bundling of microtubules can be promoted only by functional S100A8/A9 heterocomplex [27]. As S100A9 has a phosphorylation site on threonine 113 targeted by p38 MAP kinase, the effect of phosphorylation on its interactions with tubulin was also investigated [6]. Although the heterocomplex formation is not affected by the phosphorylation, both the S100A8/A9-dependent tubulin polymerization and bundling activities are abrogated, suggesting a regulatory role for phosphorylated complexes [6]. The interaction of S100A8/A9 with microtubules regulated by MAP kinase p38 and Ca^2+^-dependent signaling pathways are critical for phagocyte migration. Indeed, S100A9 −/− mice lacking functional S100A8/A9 complexes show diminished phagocyte migration and wound healing [6]. Phagocytes of S100A9 −/− mice contain significantly less polymerized as well as cytosolic non-polymerized tubulin, lower expression level and impaired activation of the small GTPases Rac and CDC42, known to be involved in migratory processes [6]. Therefore, S100A8/A9 heterocomplex is critically involved in modulation of the tubulin-dependent cytoskeleton during phagocyte migration, which provides the molecular basis for the ability of these cells to rearrange rapidly their cytoskeleton. During activation of phagocytes both proteins are released, which indicates that they may have both intra- and extra-cellular functions during inflammation [67]. The question arises of how S100A8/A9 is released into the extracellular space? Both proteins are expressed in the cytosol without any leader signal responsible for their release and they are not secreted via the classical endoplasmic reticulum/Golgi route. However, there is good evidence that active non-classical secretion and passive release from necrotic cells after tissue damage are the major physiological sources for extracellular S100A8/A9. The release of S100A8/A9 from human monocytes is energy-dependent, involves activation of protein kinase C and requires a functional microtubule network. It is associated with down-regulation of their *de novo* synthesis, suggesting that extracellular signaling via S100A8/A9 is restricted to distinct differentiation stages of monocytes [67]. The interaction of monocytes with the inflammatory activated endothelium was also described as an additional specific stimulus for S100A8 and S100A9 release [69]. It has been demonstrated that S100A8/A9 complexes secreted by activated phagocytes bind specifically to endothelial cells and directly activates the microvascular endothelium, leading to loss of barrier function, apoptosis of endothelial cells, upregulation of thrombogenic factors and an increase of junctional permeability [70,71]. These findings are further supported by observation of a massive release and interactions of S100A8, S100A9 and S100A8/A9 heterocomplexes with the endothelium in vasculitis and inflammatory arthritis, suggesting their important role in these ailments [72].

A very intriguing finding was reported recently that the S100A8/A9 tetrameric complex calprotectin is released from neutrophils as the NETs associated protein in response to infection, representing a novel unrecognized route of calprotectin extracellular release. In NETs calprotectin acts as antifungal component and its absence leads to complete loss of antifungal activity of neutrophils. By comparing the reaction in wild-type and calprotectin-deficient mice the authors have found that calprotectin is crucial for the clearance of infection, contributing to effective host defence against *Candida albicans* [73]. These findings are further substantiated by the fact that calprotectin, as a NETs associated antifungal agent, plays also role in the defense against *Aspergillus nidulans* infection as it has been shown both *in vitro* experiments and by using calprotectin knockout mice model [41].

### 6.2. Role in Inflammation and Cancer

Over the past 20 years, S100A8, S100A9 and their complexes have emerged as very potent biomarkers of a wide range of inflammatory processes, including rheumatoid arthritis, juvenile idiopathic arthritis, inflammatory bowel disease, acute lung inflammation and vasculitis [50,74,75]. Increased S100A8/A9 serum levels have been also identified as independent risk predictors for various cardiovascular diseases such as acute coronary syndrome and myocardial infarction [76–78]. S100A8 and S100A9 serve not only as useful markers of inflammation, but also play a critical role in the pathogenesis of inflammatory disorders. We have demonstrated that these proteins act as endogenous activators of TLR4 and promote inflammatory processes in infections and autoimmunity [51,52]. Thus S100A8, S100A9 and their complexes represent danger signals, which are put into action by sentinel cells sensing danger.

**Figure 3:**
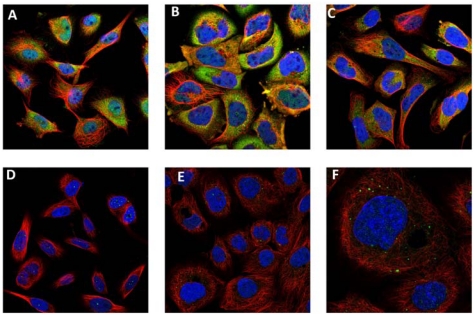
Subcellular localization of S100A8 and S100A9 in cancer cells shown by immunofluorescence [85]. Distribution of S100A9 in cytoplasm and nucleus of (**A**) U251MG glioma, (**B**) A-431 epidermoid carcinoma and (**C**) U-2 OS human osteosarcoma cell lines revealed by fluorescently labeled antibodies. Localization of S100A8 in vesicles and nucleoli of (**D**) U251MG glioma, (**E**) A-431 epidermoid carcinoma and (**F**) U-2 OS human osteosarcoma (higher magnification) cell lines revealed by fluorescently labeled antibodies. Immunostaining of S100A9 and S100A8 is shown in green, cell nucleus—in blue and microtubules—in red.

S100A8 and S100A9 play critical role in tumor biology and their elevated levels were found in numerous tumors including gastric, colon, pancreatic, bladder, ovarian, thyroid, breast, skin and prostate cancers [4,79–84]. They are abundantly expressed in neoplastic cells and also in infiltrating immune cells [79–81,83,84]. By using immunostaining the expression of S100A8 and S100A9 are shown in the tumor cells such as U251MG glioma, A-431 epidermoid carcinoma and U-2 OS human osteosarcoma cell lines (Figure 3). The different level of staining for S100A8 and S100A9 may reflect the individual variability in their expression level.

S100A8 and S100A9 play pathogenic roles in cancer progression in a concentration-dependent manner. At low concentrations S100A8/A9 complexes promote tumor cell growth [86,87] and tumor cell migration [2,82,88], while at high concentrations apoptotic effects on tumor cells were observed [86]. Importantly, intracellular S100A8/A9 proteins regulate the accumulation of myeloid-derived suppressor cells [89–91]. These are immature myeloid cells that expand during inflammation and in tumors, and act as potent suppressors of T-cell mediated immune responses [92]. One of the major immunological abnormalities in cancer is the accumulation of myeloid-derived suppressor cells leading to inhibition of dendritic cell differentiation and suppression of antitumor immune responses.

Cheng *et al.* [89] showed that tumor derived factors promote sustained Stat3-dependent upregulation of S100A9 in myeloid precursors, which results in inhibition of differentiation of dendritic cells and accumulation of myeloid-derived suppressor cells. Mice lacking S100A9 rejected implanted tumors. These findings strongly suggest that the S100A8/A9 proteins support an autocrine feedback loop that sustains accumulation of myeloid-derived suppressor cells in tumors [89,93]. Similar results were obtained by Ichikawa and co-authors on colon tumors, where in mice lacking S100A9 the authors observed significantly reduced tumor incidence, growth and metastasis, decreased chemokine levels and declined infiltration of CD11b^+^ Gr1^+^ cells within tumors and premetastatic organs [91]. These studies demonstrated that tumor-induced up-regulation of S100A9 protein is critically important for accumulation of myeloid-derived suppressor cells and revealed a novel molecular mechanism of immunological abnormalities in cancer.

### 6.3. Role in Signaling Cascades

Important pro-inflammatory roles of S100A8 and S100A9 in numerous processes are supported by the abundant findings of their high local expression levels and the plentiful effects produced on various cell types. However, the exact molecular signal transduction mechanisms and specifically their interactions with different receptors such as TLR4 and RAGE are still a matter of debate. We have recently demonstrated, that S100A8/A9 complexes are endogenous activators of TLR4 and via this signaling pathway they promote inflammatory processes in infections and autoimmunity [5,51,52]. In contrast, mice deficient in S100A8/A9 complexes were protected from endotoxin induced septic shock and *E. coli* induced abdominal sepsis. We demonstrated that S100A8/A9 heterodimer and S100A8 homodimers bind to and signal directly via the lipopolysaccharide receptor complex including TLR4, MD2 and CD14. S100A8 is the active component, which specifically binds to TLR4 and this consequently induces the recruitment of the adaptor protein MyD88, activation of IRAK-1, MAP kinases ERK1/2 and p38. After activation and translocation of the transcription factor NF-κB, the cytokines and chemokines become released, which contribute significantly to the overshooting immune response during sepsis [51,94].

These were the first proofs of cell activation by S100A8/A9 via TLR4 cascade, where the S100A8/A9 proteins fulfill the function of a danger associated molecular pattern (DAMP) molecule. DAMPs are endogenous molecules or alarmins released either after cell activation or by necrotic cells, but not by apoptotic cells. When released these endogenous molecules are able to recruit and activate numerous cell types of the innate immune system expressing DAMP receptors such as Toll-like receptors 2, 4 and 9 or RAGE, thereby amplifying inflammatory reactions [95]. In experimental mouse models of arthritis the pro-inflammatory and TLR4 dependent activities of S100A8/A9 were further confirmed. S100A8 mediates cartilage destruction, chondrocyte death and osteoclastic bone resorption [1,5,53]. In addition in human and mouse osteoarthritis both S100A8 and S100A9 affect chondrocyte activation and joint destruction also acting via the TLR4 signaling cascade [96–98].

It is important to note, that in various inflammatory autoimmune disorders both S100A8 and S100A9 are among those proteins whose expression is most up-regulated [50,99,100]. Recently we demonstrated a direct link between local S100A8/A9 expression and the development of systemic autoimmunity. In a mouse model of autoimmune disease autoreactive CD8+ T cells were induced after the local release of S100A8/A9, resulting in the development of systemic autoimmunity. This effect was again mediated via TLR4 signaling pathway leading to increased interleukin-17 expression and the development of autoreactive lymphocytes [52]. This newly identified connection between the local production of S100A8/A9 as DAMP molecules and autoimmunity may have direct implications for many other chronic inflammatory diseases.

Apart from TLR4, S100A8/A9 proteins bind to RAGE [82,86,87,101,102]. Specifically, in human prostate cancer cells Hermani *et al*. found a strong co-localization of intracellular S100A8/A9 with RAGE after stimulating cells by adding recombinant S100A8/A9 proteins or by increasing cytosolic Ca^2+^ level [82]. Ghavami *et al*. showed that S100A8/A9 complexes promote growth of various tumor cells through RAGE signaling and activation of NF-κB [86]. The interaction of S100A8/A9 with RAGE and their effect on post-receptor signaling was identified also in cardiomyocytes [102]. Large increases in the levels of S100A8 and S100A9 was observed in cardiomyocytes and whole hearts exposed to systemic lipopolysaccharide. Consequently, in the intact mice a decreased cardiac ejection fraction was observed, whereas S100A9-knockdown mice attenuated lipopolysaccharide-induced cardiac dysfunction. Thus lipopolysaccharide-induced expression of S100A8 and S100A9 led to a RAGE-dependent decrease in calcium flux and a RAGE-mediated decrease in cardiomyocyte contractility.

It is important to note, that in many of these systems both TLR4 and RAGE are present on the same cell or cell line, however, most likely only one receptor is used for extracellular S100A8/A9 signaling at the same time [1,52,87,91,97,98]. It remains to be identified how cellular environment, pathophysiological conditions as well as the structural properties and oligomeric states of S100A8 and S100A9 can affect the interaction of these proteins with specific receptors.

## 7. Amyloid Complexes

There is increasing evidence that S100 proteins are able to form higher-order oligomers [14,103–105], though the functional relevance of these complexes is not yet clear. Recently we have found that S100A8/A9 can self-assemble into a variety of amyloid complexes both *in vivo* and in the test tube *in vitro*. In the human body they are involved in the formation and deposition of amyloids in the ageing prostate known as corpora amylacea inclusions [15]. This is a type of localized amyloidosis, progressing with ageing and frequently occurring in the middle-aged and older men. Clinically corpora amylacea inclusions are often linked to asymptomatic prostate inflammation and localized adjacent to the damaged epithelium and focal inflammatory infiltrates [106–108]. Corpora amylacea deposits were detected also in up to 55% specimens with high-grade prostatic intraepithelial neoplasia derived from radical prostatectomies [109]. As inflammation plays a crucial role in prostate pathogenesis and was linked to 40–90% benign prostatic hyperplasia [110] as well as with 20% of all human cancers [106,111], prostate corpora amylacea can be viewed as the risk factor for cancer development [15]. Indeed, prostate cancer is the most common non-cutaneous malignant neoplasm in men in Western countries, affecting several millions men, and its incidence is rising rapidly with ageing population. Therefore the cancer risk assessment is of critical significance in its preventing strategies.

On the other hand, the amyloid component of corpora amylacea is closely related to insoluble amyloid structures of other functional proteins and peptides that are deposited in variety of tissues and organs, losing their original function and acquiring gain-on functions such as amyloid cytotoxicity. These protein self-assembling complexes and their deposits serve as a hallmark of growing number of age-related degenerative ailments, including neurodegenerative Alzheimer’s and Parkinson’s diseases, type II diabetes, systemic and localised amyloidoses and others. To date about 30 different polypeptides with completely unrelated primary, secondary and tertiary structures are known to be involved in the amyloid disorders, all developing typical for amyloid fibrils cross-β sheet rich core [112]. It is important to note, that in the ageing prostate such fundamental but seemingly different processes as degeneration, inflammation and cancer are closely inter-connected via common key element—the amyloid formation in corpora amylacea.

The amyloid-containing corpora amylacea inclusions can significantly vary in dimensions, growing from sub-millimetre to a few millimetres diameters and constituting in their bulk up to a third of the prostate gland weight in some clinical cases. Up till now they often were viewed simply as calcified bodies, prostatic concretion or calculi, resulting from calcification of precipitated prostatic secretion [113,114] or arising from simple precipitation of salts normally presented in prostatic fluid [115]. However, as revealed by X-ray photoelectron spectroscopy, Fourier transform infrared spectroscopy and thermogravimetry the proteinaceous component constitutes up to 30–40% of corpora amylacea weight, while the rest corresponds to inorganic compounds consisting of hydroxylapatite (Ca_5_(PO4)_3_OH) and whitlockite (Ca_2_(PO4)_3_) and containing high concentration of zinc.

**Figure 4:**
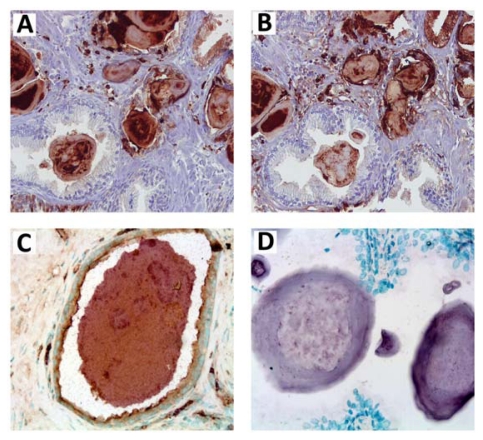
Localization of S100A8 and S100A9 proteins and their amyloids in prostate corpora amylacea and surrounding tissues revealed by immunohistochemical analysis. Immunohistochemistry of prostate corpora amylacea and surrounding tissues with (**A**) anti-S100A8 and (**B**) anti-S100A9 antibodies, (**C**) co-immunostaining of corpora amylacea with anti-S100A8 (purple) and anti-S100A9 (brown) antibodies; (**D**) immunostaining of corpora amylacea with generic anti-fibrillar antibodies. Magnification is 80× in (**A**,**B**), 100× in (**C**) and 200× in (**D**). Adapted from Yanamandra *et al*. [15].

**Figure 5:**
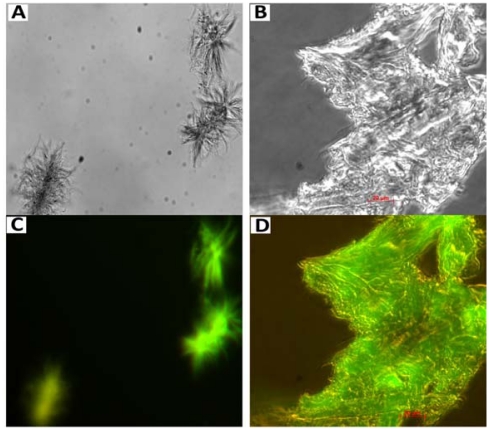
Prostate corpora amylacea extracts stained with amyloid specific dye—thioflavin-T. Optical bright field images (**A**,**B**) and related fluorescence images, showing intensive thioflavin-T fluorescence (**C**,**D**). Scale bar is 20 μm [118].

By using mass-spectrometry, gel electrophoresis and Western blot analyses, the pro-inflammatory S100A8/A9 were found to be a major proteinaceous component of all corpora amylacea specimens obtained as a result of prostatectomy of the prostate cancer patients [15]. Immunohistochemical analysis revealed that the corpora amylacea are uniformly stained with antibodies against both S100A8 and S100A9 (Figure 4). Positive staining with anti-S100A8 and anti-S100A9 antibodies was observed also in the epithelial tissues adjacent to corpora amylacea, infiltrated with the tissue macrophages. As macrophages are the source of S100A8 and S100A9, this can lead to a significant increase of the local S100A8/A9 content, which can serve as a risk factor for the concentration-dependent amyloid formation. Remarkably, all corpora amylacea inclusions were also stained with anti-amyloid fibril antibodies [116], (Figure 4D), as well as with thioflavin-T and Congo red dyes, used as specific markers for the amyloid depositions (Figure 5). All these observations demonstrate that the amyloid material constitutes a significant mass of corpora amylacea. The calcification of amyloid deposits occurring in the prostate lead to their further stabilization, which is particularly important in the protease rich prostate fluid. Indeed, the mineral content of corpora amylacea is rather uniform in all studied patients, indicating that calcification can be a regulated process. It is important to note, that S100A9 itself can serve as a promoter of dystrophic calcification process as it has been shown in atherogenesis [117].

**Figure 6:**
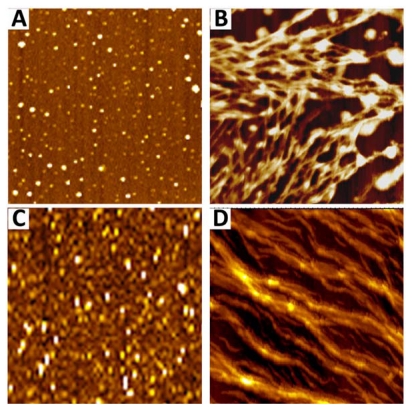
AFM images of S100A8/A9 amyloid oligomers and fibrils extracted from prostate corpora amylacea (**A**,**B**) and produced *in vitro* (**C**,**D**), respectively. Size of images is 4 × 4 μm. Adapted from Yanamandra *et al*. [15].

Furthermore, the amyloid aggregates were extracted from corpora amylacea and atomic force and transmission electron microscopy analyses revealed that the extracts contain a variety of highly heterogeneous aggregates (Figure 6), from oligomeric species to extensive networks of mature fibrils and larger scale super-molecular assemblies, reaching a few microns in length. The amyloids of S100A8/A9 were produced also *in vitro* to match the *ex vivo* species and provide further insight into their structural properties. Highly heterogeneous S100A8/A9 amyloid aggregates were produced from both recombinant proteins expressed in *Escherichia coli* and those extracted from granulocytes, which were both incubated under the native conditions of pH 7.4 and 37 °C with agitation as well as at pH 2.0 and 57 °C without agitation [15]. At pH 7.4, the species resembling *ex vivo* oligomers and short protofilaments were formed after 2 weeks and very thick bundles of fibrils with heights of 15–20 nm in the atomic force microscopy (AFM) cross-sections and a few microns in length constituted the major population of fibrillar aggregates after 8 weeks of incubation (Figure 6). After 4 week incubation at the acidic pH the S100A8/A9 were assembled into the flexible fibrils with height of *ca.* 4–5 nm in the AFM cross-section and microns in length together with straight, rigid but rather short fibrils of a few hundred nanometers in length, both of which closely resembling the *ex vivo* species. Calcium and zinc played a critical role in promoting the S100A8/A9 amyloid formation *in vitro*.

Indeed, the S100A8/A9 amyloid protofilaments of *ca.* 2 nm height and thicker mature fibrils were assembled in the presence of 10 mM ZnCl_2_ and in a suspension of Ca_3_(PO_4_)_2_ [15], but not when EDTA was added in solution. As the *ex vivo* corpora amylacea deposits are calcified and contain much zinc salts, these metals can play a critical role in S100A8/A9 amyloid formation *in vivo*. The bundles of amyloid fibrils of S100A8/A9 proteins, formed *in vitro* and extracted from *ex vivo* material [15], are amongst the largest reported amyloid super-molecular species. The lateral association and thickening of the fibrils is likely to be a contributing factor to their stability in the prostate gland. It has been suggested that the various functions of the S100A8/A9 hetero- and homooligomers may be regulated, apart from all other mechanisms discussed above, also by their differential protease sensitivity [119]. The heterooligomeric complexes of S100A8/A9 are characterized by significant stability and protease resistance comparable to these of prions. In the protease rich environment of prostate gland, and especially at sites of inflammation, where proteases are present at even higher levels, protease resistance of the S100A8/A9 proteins could favour their accumulation and conversion into amyloid structures. If so, the amyloid structures formed by S100A8/A9 can be at the extreme end of the scale of resistance to proteolysis.

It is important to note, that the calculated intrinsic aggregation propensity scores of monomeric S100A8 and S100A9 at both pH 7.0 and pH 2.0, the conditions of their *in vitro* amyloid formation, are rather high and comparable to those of Aβ peptides, forming amyloid deposits in Alzheimer’s disease [15]. The overall aggregation scores for S100A8 are equal to 0.76 and 0.77, while for S100A9 they are equal to 1.04 and 0.65 at pH 7.0 and pH 2.0, respectively. By comparison, for Aβ (1–40) and Aβ (1–42) peptides at pH 7.0 the scores are 0.97 and 0.94, respectively. In both proteins the Ca^2+^-binding sites are located in close proximity to the segments that are highly aggregation-prone. However, in the S100A8/A9 heterodimer the amyloid scores for S100A8 and S100A9 are significantly reduced and equal to 0.18 and 0.32, respectively, indicating that most of the aggregation-prone sequences are involved in the native complex formation. The native-like assembly of three heterotetramer calprotectin molecules is shown in Figure 2E, which highlights how the native conformation can evolve into the larger oligomers. It is likely that Ca^2+^-dependent native complex formation can effectively compete under physiological conditions with the Ca^2+^-dependent amyloid assembly and the latter may be prevalent in destabilizing environment leading to native complex dissociation.

As prostatic fluid is very rich in protein content, small quantities of other proteins were also found in the corpora amylacea inclusions, presumably being trapped in the aggregating and growing deposits. Among them, the finding of *Escherichia coli* DNA and *Escherichia coli* proteins indicates that corpora amylacea formation may be associated with bacterial infection, causing consequently inflammation in surrounding tissues during the course of corpora amylacea initiation and growth. The identification of highly amyloidogenic bacterial co-chaperonin GroES can be related not only to the fact that bacterial infection is a contributory factor to inflammation, but suggests also the potential role of bacterial infection in the initiating of the amyloid depositions via amyloid seeding [120].

Thus the amyloid growth in the ageing prostate can be promoted by the self-perpetuating cycle triggered by the inflammation and the increasing concentration of aggregation-prone S100A8/A9 proteins in the inflammatory sites. This would favour the amyloid assembly and deposition, as amyloid formation is a concentration-dependent process. The abundance of calcium and zinc salts in corpora amylacea can further promote this process, especially as S100A8/A9 can themselves regulate their own calcification [117]. In the course of corpora amylacea growth, neighboring acini are obstructed, exacerbating inflammation and enhancing the risk of neoplastic transformation. It is interesting to note, that the amyloid propensity of other S100 proteins, such as S100A3, S100A6, S100A12 and S100B, was evaluated recently by computational analysis [7]. It was shown that they all contain amyloid-prone areas, therefore all can access the amyloid conformation under particular physiological conditions and metal ions can play role in this process. In the near future we may expect the discovery of the amyloids of other S100 proteins both *in vitro* and *in vivo* under closer investigation. It is apparent that the native and amyloid oligomerization processes of S100A8 and S100A9 represent competing pathways which further diversify their structural and functional properties.

## 8. Conclusions

The direct involvement of pro-inflammatory S100A8/A9 proteins in corpora amylacea biogenesis emphasizes the role of inflammation and amyloid formation in the age-dependent prostate remodeling and cancerogenesis. As the enhanced level of S100A8 and S100A9 proteins is a characteristic feature of numerous inflammatory, cancers and degenerative conditions taking place in different tissues and organs, they may effectively contribute to the disease pathologies also via amyloid depositions. Both proteins are characterized by high intrinsic amyloid propensity and the effect on their amyloid self-assembly of various environmental factors requires thorough investigation. This fact may add to the complexity of the pathological process in question, but if properly understood, S100A8, S100A9 and their complexes can be used as potent therapeutic targets in a wide range of human ailments.
